# Early detection of variants of concern via funnel plots of regional reproduction numbers

**DOI:** 10.1038/s41598-022-27116-8

**Published:** 2023-01-19

**Authors:** Simone Milanesi, Francesca Rosset, Marta Colaneri, Giulia Giordano, Kenneth Pesenti, Franco Blanchini, Paolo Bolzern, Patrizio Colaneri, Paolo Sacchi, Giuseppe De Nicolao, Raffaele Bruno

**Affiliations:** 1 Department of Mathematics, University of Pavia, Pavia, Italy; 2 Department of Mathematics, Computer Science and Physics, University of Udine, Udine, Italy; 3 Division of Infectious Diseases I, Fondazione IRCCS Policlinico San Matteo, Pavia, Italy; 4 Department of Industrial Engineering, University of Trento, Trento, Italy; 5 Department of Surgical Medical and Health Sciences, University of Trieste, Trieste, Italy; 6 Department of Electronics, Information and Bioengineering, Politecnico di Milano, Milan, Italy; 7 Institute of Electronics, Information Engineering and Telecommunication (IEIIT), Italian National Research Council (CNR), Turin, Italy; 8 Department of Electrical, Computer and Biomedical Engineering, University of Pavia, Pavia, Italy; 9 Department of Clinical, Surgical, Diagnostic, and Pediatric Sciences, University of Pavia, Pavia, Italy

**Keywords:** Epidemiology, Scientific data, Statistics

## Abstract

Early detection of the emergence of a new variant of concern (VoC) is essential to develop strategies that contain epidemic outbreaks. For example, knowing in which region a VoC starts spreading enables prompt actions to circumscribe the geographical area where the new variant can spread, by containing it locally. This paper presents ‘funnel plots’ as a statistical process control method that, unlike tools whose purpose is to identify rises of the reproduction number (${R}_{t}$), detects when a regional ${R}_{t}$ departs from the national average and thus represents an anomaly. The name of the method refers to the funnel-like shape of the scatter plot that the data take on. Control limits with prescribed false alarm rate are derived from the observation that regional ${R}_{t}$'s are normally distributed with variance inversely proportional to the number of infectious cases. The method is validated on public COVID-19 data demonstrating its efficacy in the early detection of SARS-CoV-2 variants in India, South Africa, England, and Italy, as well as of a malfunctioning episode of the diagnostic infrastructure in England, during which the Immensa lab in Wolverhampton gave 43,000 incorrect negative tests relative to South West and West Midlands territories.

## Introduction

All viruses, including SARS-CoV-2, evolve over time. Mutations happen frequently and, in most cases, have little to no impact on the viral function. However, a group of mutations with similar genetic lineage, denoted by public health organizations as Variants of Concern (VoC), have gained global attention because of their faster spread and evidence for higher transmissibility and possibly higher virulence^1^.

Surveillance aimed at the early detection of a new VoC is fundamental. The World Health Organization (WHO) and its international networks of experts closely monitor SARS-CoV-2 variants^2^, but a surveillance system at a national and sub-national level is crucial to identify the emergence of new variants with the potential to spread worldwide, as well as the spread of already detected variants. Local authorities are thereby currently encouraged to strengthen surveillance and sequencing capacities, to early detect unusual epidemiological events. However, several countries still have limited capacity, despite the enormous efforts to facilitate the access to existing international networks^3^ and the implementation of low-cost whole genome sequencing (WGS) methods^4^.

As happened with SARS-CoV outbreaks^5^, new SARS-CoV-2 variants with unforeseen mutations continue to emerge^6–8^, also with the potential risk of immune evasion^9,10^. The Omicron variant (B.1.1.529 lineage), which contains over 30 mutations in the spike protein, including the same mutations of pre-existing VoC, will definitely not be the last, and possibly not the most challenging we will ever face^11^. The important task of designing early warning systems requires a panoplia of tools, ranging from genome sequencing, epidemiological surveillance, and machine learning applied to spike protein mutations^12,13^.

To support monitoring based on epidemiological data, we propose a statistically based methodology that is easy to apply and enables the early detection of anomalous events, consequently triggering further inquiries. With respect to massive genomic sequencing, statistical methods based on epidemiological data are faster and reduce costs and needed resources; of course, they do not replace sequencing, but integrate it and may defer the genomic sequencing methods to a more targeted and purpose-driven framework, to effectively detect potential VoCs and prevent their spread.

The keystone of our approach is the use of statistical quality control to monitor the homogeneity of the time-varying estimated reproduction numbers of the disease in different regions of a country (or, more in general, in different geographical areas).

The novelty of the approach consists in statistically comparing the reproduction numbers of different regions in order to detect if some territories behave as outliers. As a key feature of the proposed methodology, a rigorous statistical threshold is derived, which accounts for the different sample sizes, i.e., the number of infectious cases in a region. In the general context of healthcare monitoring, this sample size issue had come under the spotlight in the early 2000, in a series of works^14–16^. An example was the detection of abnormal mortality rates in cardiac surgery wards^14^: through the characterization of the baseline variability, one could build control charts with statistical limits which, if exceeded, suggested the existence of an abnormal cause explaining the anomalous mortality. When the key performance indicators were affected by the sample size, it was shown that their monitoring could rely on so-called funnel plots^17,18^.

In the case of epidemics, anomalies can be detected by a comparative monitoring of the regional effective reproduction number, ${R}_{t}$, whose variance depends on the number of new infected subjects in the given region. Closely related to ${R}_{t}$ is the so-called basic reproduction number ${R}_{0}$ (i.e. ${R}_{t}$ at the beginning of the epidemic outbreak) whose expression is obtained from mathematical models. For the analytical and numerical computation of ${R}_{0}$ for general structured population models, see^19–21^.

A large regional ${R}_{t}$ may have a special cause, such as the emergence of a new VoC, or may just be the effect of statistical fluctuations due to sampling noise. In this work, to monitor the onset of statistical anomalies in regional ${R}_{t}$’s, we derive suitable funnel plots whose control limits can reveal abnormal trends, while keeping false alarms under control. We validate our proposed methodology using publicly available epidemiological data from Italy, England, India and South Africa: we show that the crossing of control limits promptly reveals the emergence of new more transmissible variants or the malfunctioning of the diagnostic infrastructure.

In conclusion, we notice that the utility of funnel plots is not limited only to epidemiological setting, but have also meaningful clinical implications. Indeed, several papers^22,23^ show that VOCs have a reduced sensitivity to both antiviral drugs and monoclonal antibodies. The capacity to detect VOCs earlier means the possibility to improve the appropriateness of early therapies and to reduce hospitalizations and deaths.

## Results

We apply the funnel plot methodology to five case studies, corresponding to different stages of the COVID-19 pandemic, chosen because of their relevance to the spread of VoC's or to flaws of the diagnostic infrastructure. Two case studies refer to England (initial spread of the Omicron variant in December 2021 and large failure of a diagnostic lab in September 2021), and the other three to Italy (initial spread of the Omicron variant in December 2021), India (first emergence of the Delta variant in February 2021), and South Africa (first emergence of the Omicron variant in November 2021). In addition, the nine English regions are monitored over a 18-month period from December 2020 to June 2022.

In all cases, we focus on four key dates. The first date corresponds to a situation of statistical homogeneity: when variants are uniformly spread in the country and contact rates do not vary much across regions, differences between estimated ${R}_{t}$'s are exclusively due to natural variability and the regional ${R}_{t}$’s are expected to lie within the funnel, centered around the national ${R}_{t}$ (see Methods). The second and the third dates refer to the disruption of the natural variability: when a new VoC starts spreading, at first it colonizes in particular a few territories, whose behavior becomes abnormal with respect to the national one. This is highlighted by the fact that the corresponding ${R}_{t}$’s first cross the funnel limits and then clearly move outside the limits. The last date corresponds to a new homogeneity, typically established around a higher ${R}_{t}$: the VoC is now uniformly spread in the country, thus restoring the condition of natural variability. Finally, to have a snapshot of the whole period under study, the standardized ${R}_{t}$'s with ± 3.09 sigma are plotted on a Bonferroni control chart, which is a standard univariate control chart whose control limits are adjusted according to the Bonferroni correction (see Methods for details). Due to its statistical background, the scope of the new control method is not restricted to VoC monitoring, but can detect other kinds of anomalies, such as those related to testing availability or malfunctioning buffer factories: we discuss an example of such an anomaly in our Immensa case study.

Hereafter, the infectious cases at day t are the total number of individuals that are infected and infectious at day t, while the new cases at day t are the number of subjects who become infectious at that time.

### Spread of the Omicron variant in Italy

We first apply the funnel plot methodology to the Italian regional data in the period from 4 December to 3 January 2022, based on epidemiological indicators released daily by the Civil Protection Department, which provides 21 regional time series (for 19 regions and the 2 autonomous provinces of Trento and Bolzano). The Delta variant was dominant in Italy until December 2021, when the Omicron variant started to spread across the country.

**Figure 1:**
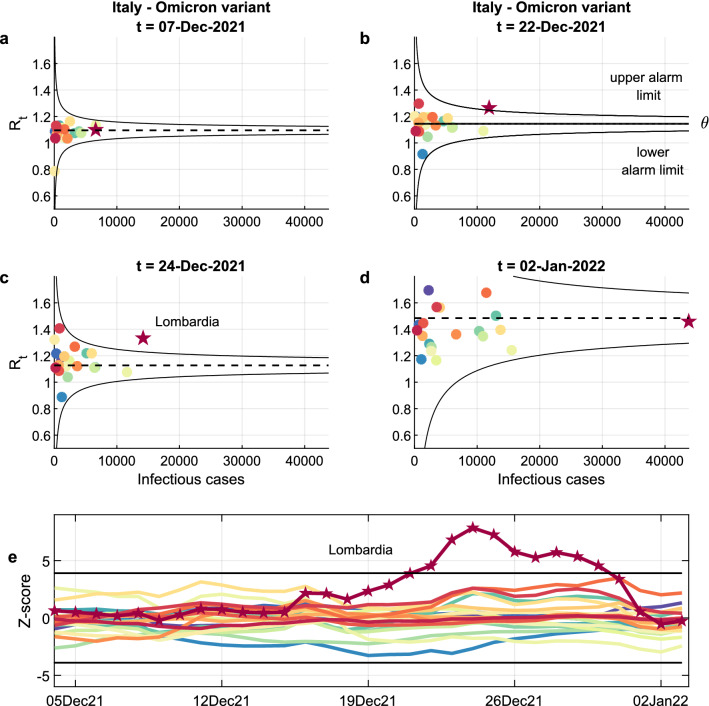
Monitoring regional reproduction numbers (R_t_’s): funnel plots and control chart. Panels **a**-**d** show the Italian regional R_t_’s (colour-coded circles, see Supplementary {1} for the legend), plotted against the infectious cases at four selected times. When the epidemic evolution is homogenous across regions, differences between R_t_’s are exclusively due to natural statistical variability and the circles are expected to lie inside the black alarm limits in 99.8% of the cases. The alarm limits have the shape of a funnel because the variance of the estimated R_t_ is inversely proportional to the number of infectious cases. The central dashed line represents the average R_t_. A circle is out of statistical control if it lies outside the black funnel. Out-of-control circles might therefore reveal anomalies that disrupt the homogeneity between regions. In (**a**–**d**), the majority of the points, lying in the funnel, are essentially indistinguishable and therefore not even named. On 22 December 2021, Lombardia (dark red) crossed the alarm limit and on 24 December 2021 it was completely outside the upper alarm limit. As confirmed by a survey by the Italian National Institute of Health, Lombardia was the first Italian region to be colonized by the Omicron variant. As the other regions were colonized too, the distribution of their R_t_’s moved upward and, on 2 January 2022, Lombardia was again inside the funnel. The trend can be monitored by plotting the standardized R_t_’s on a Bonferroni control chart with $\pm 3$.09 (**e**), where the introduction of the Omicron variant in Lombardia in mid-December is clearly visible.

The results are summarized in Fig. 1. In the Panels a-d, the estimates of Italian regional ${R}_{t}$'s are plotted against the infectious cases on four selected dates. On 7 December 2021 (see panel a), differences between estimated ${R}_{t}$'s were due to natural variability alone and the 21 points lay within the funnel limits. On 22 December 2021, Lombardia (dark red) crossed the alarm limit (see panel b) and on 24 December 2021 (see panel c) it was definitely outside the upper alarm limit. In fact, as confirmed by a retrospective survey by the Italian National Institute of Health published on 31 December 2021^24^, Lombardia was the first Italian region to be colonized by the Omicron variant. As other regions became increasingly colonized by the Omicron variant, their ${R}_{t}$'s rose as well and, by 2 January 2022, Lombardia was absorbed again within a funnel, now with a higher mean than in early December (see panel d).

We can monitor the trend by plotting the standardized ${R}_{t}$'s on a Bonferroni control chart with ± 3.09 sigma limits (see panel e), where the arrival of the Omicron variant in Lombardia in mid-December is clearly detectable.

### Statistical monitoring of England for a year and a half

**Figure 2:**
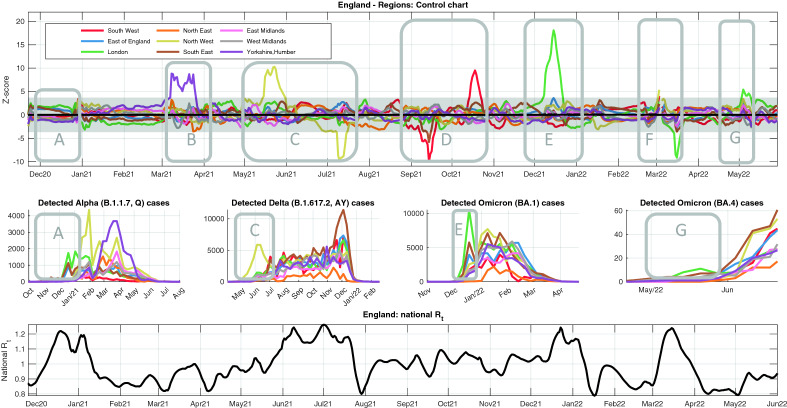
Monitoring regional homogeneity in England from December 2020 to May 2022. The upper panel shows the Bonferroni control chart of the normalized R_t_’s of English regions during 18 months. In this period, seven major events, labelled from A to G, are visible, whose plausible explanations are conjectured below. A– Alpha variant: although no crossing is observed, the curves form two clusters, and the upper is formed by the three regions where the alpha variant first became dominant. No crossing occurs because, rather than starting in a unique region, the variant colonized three regions at the same time, hindering out-of-control detection. B–Outbreaks in Yorkshire: during Spring 2021, the number of infection cases in the region was slowly decreasing and then it had a huge peak, due to very high numbers of manufacturing jobs and related high-exposure workplaces, leading to outstanding outbreaks such as the one in a Selby warehouse with more than 700 employees. C– Delta variant first arrival in the North West: in May 2021 the Delta variant, originated in India, started its colonization of England from the North West region. As a characteristic feature of the control chart, the curve that first crosses the upper limit in correspondence of a new VoC, later on is often going to cross also the lower limit if its R_t_ is the first one to decrease, as happened to the North West. D– Immensa scandal: see Fig. 4 for a discussion of the massive lab malfunctioning that perturbed case recording in the South West and South East. Compared to the rise of a new VoC, a specular pattern is observed: when the malfunctioning is fixed, a fake outbreak is observed and the red curve crosses the upper control limit. E – Omicron 1 variant first arrival in the London region: see Fig. 3k–o for a discussion. F – In mid February, the UK Government announced that on April 1 free tests would be suspended, thus triggering social behaviors that may explain the anomalous trends in the control chart. G – Omicron 4 variant first arrival in the London region. For validation purposes, the four middle panels report the number of weekly detected cases, in each region, of the main VoCs^36^, Alpha (B.117, Q) , Delta (B.1.617.2, AY), Omicron 1 (BA.1) and Omicron 4 (BA.4), during selected time windows. In each case, the control chart correctly identifies the region where the spread starts. The number of detected cases for Omicron 4 is far less than for the other VoCs, due to cuts to genomic surveillance: the value of the control chart as a surveillance tool is even more evident. Finally, the bottom panel reports the time profile of the national R_t_, to facilitate the connection with the different phases of the pandemic in UK.

Figure 2 displays the Bonferroni control chart of normalized ${R}_{t}$'s of the nine English regions during 18 months, from the end of November 2020 to the beginning of June 2022. Under natural variability conditions, irrespective of the current national ${R}_{t}$, all the normalized curves are expected to lie within the limits. Points outside the limits highlight a disruption of the statistical homogeneity across regions, which should be investigated to unveil the root cause of the anomaly. Figure 2 reports seven major events, labelled from A to G, along with plausible conjectured explanations: the emergence or the arrival of the VoCs (Alpha^25,26^, Delta^27^, Omicron^28,29^ and Omicron sub-variants^11^), the malfunction of swab factories (further analyzed in Fig. 4)^30,31^, some incidents of violation of lockdown restrictions^32–34^, and changes in the testing policies^35^.

**Figure 3:**
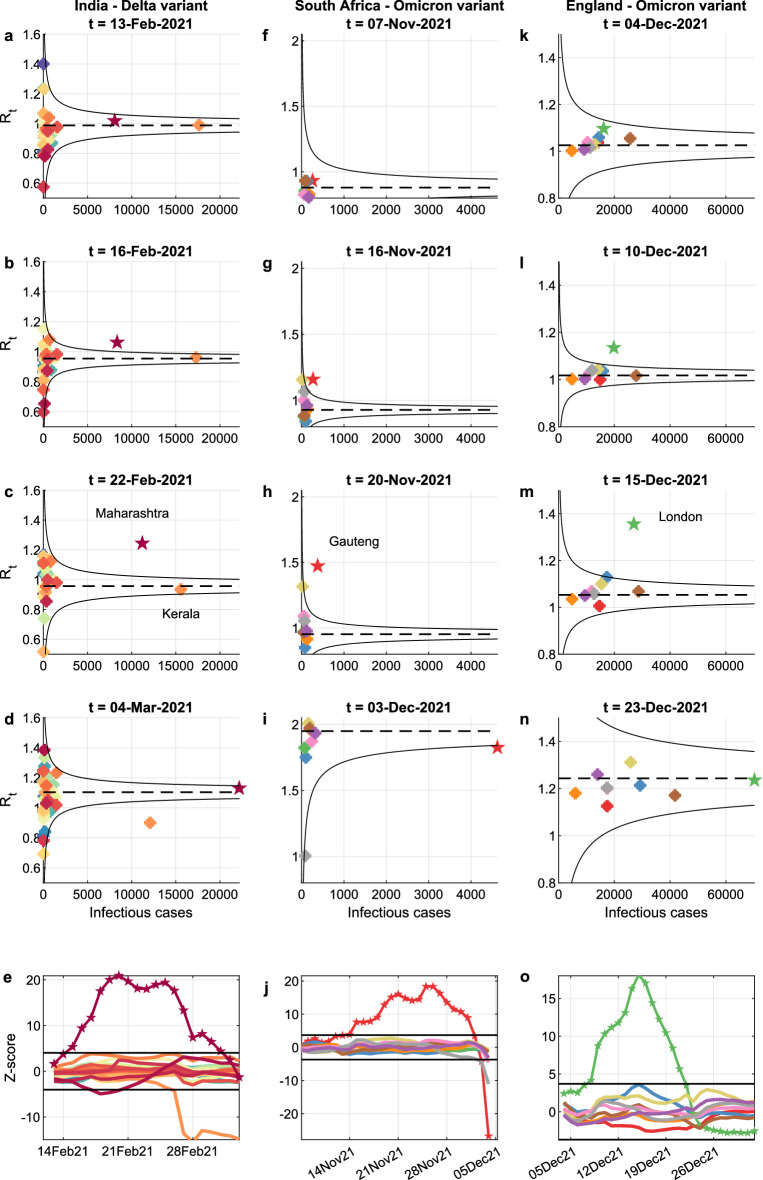
Funnel plots help detect anomalies: spread of the Delta variant in India and of the Omicron variant in South Africa and England. India: (**a**–**d**) display the funnel plots at four selected times, with colour-coded circles corresponding to the R_t_’s of the Indian states. On 13 February 2021, all points are within the funnel, but on 16 February 2021, when the Delta variant starts spreading, there is an out-of-control point corresponding to Maharashtra (dark red), which on 22 February 2021 is further apart from the mean. Finally, on 4 March 2021 the R_t_’s of all regions except Kerala (orange) converge to a new distribution characterized by a higher R_t_. The trend can be monitored by plotting the standardized R_t_’s on a Bonferroni control chart with $\pm 3$.09 sigma limits, see Panel **e**, where the rise of the Delta variant in Maharashtra is clearly visible. South Africa: (**f**–**i**) display the funnel plots at four selected times, with colour-coded circles corresponding to the R_t_’s of the South African provinces. The rise of the Omicron variant in the Gauteng province (red) is well visible both in the funnel plots and in the Bonferroni control chart reported in (**j**). England: (**k**–**n**) display the funnel plots at four selected times, with colour-coded circles corresponding to the R_t_’s of the English regions. The spread of Omicron in England started from the London region (green), whose R_t_ had already crossed the alarm limit on 10 December, when, as seen in Fig. 3E of^29^, the daily proportion of Omicron infections did not exceed 25%. As the other regions were colonized, the distribution of their R_t_ moved upward and, on 23 December, the London region was again inside the funnel, as also seen in the Bonferroni control chart reported in Panel (**o**).

**Figure 4:**
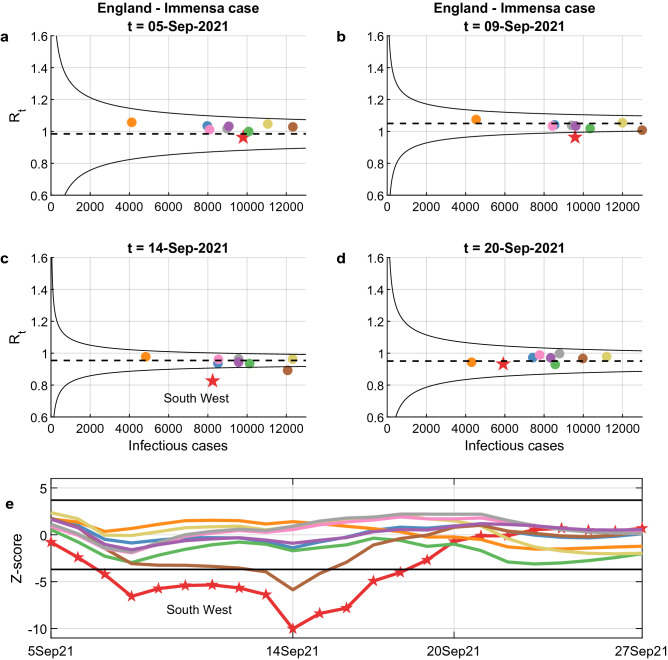
Funnel plots help detect anomalies: the incorrect negative tests of the Immensa lab in England. (**a**–**d**) display the funnel plots at four selected times, with colour-coded circles corresponding to the R_t_’s of the England regions. On 5 September 2021, all circles were inside the funnel, but on 9 September 2021 there was an out-of-control point below the lower alarm limit corresponding to South West (red), which was further apart from the mean on 14 September 2021, when also West Midlands (brown) went below the lower limit. Finally, on 20 September 2021 the R_t_’s of all regions returned within the limits. The anomalous decrease of R_t_ in the South West corresponds to the period during which the Immensa lab (Wolverhampton) gave some 43,000 incorrect negative tests relative to South West and West Midlands. The whole trend can be monitored by plotting the standardized R_t_’s on a Bonferroni control chart with  $\pm \hspace{0.17em}3.09$ sigma limits, (**e**). Lab operations were suspended in mid-October as a consequence of the malfunction, while the control chart indicated an out-of-control condition as early as late August.

### Emergence of the Delta variant in India

We applied our methodology to epidemic data from India in the period 13 February–5 March 2021, when the Delta variant emerged and started spreading from the state of Maharashtra. Panels a-d of Fig. 3 show funnel plots at four selected times, where colour-coded circles represent the ${R}_{t}$’s of the 36 Indian states. While on February 13 all circles fell within the funnel, on February 16 the state of Maharashtra (dark red) crossed the alert threshold (in correspondence with the initial spread of the Delta variant), further departing from the mean on February 22. Lastly, on 4 March 2021, the ${R}_{t}$’s of all regions but Kerala (orange) shaped a new funnel with a higher mean, which again incorporated Maharashtra. The peculiar dropping of Kerala’s ${R}_{t}$ below the lower alert threshold, despite the very high number of infectious cases, might be explained by the co-circulation of Alpha and Delta variants during the same period, resulting in a lower ${R}_{t}$ than in the areas predominantly hit by the Delta variant.

In the Bonferroni control chart (see panel e), the rise of the Delta variant in Maharashtra is clearly visible since mid-February 2021. One month later, on March 17, it was disclosed that a 10-lab research consortium had alerted the Union Health Ministry about a new variant spreading in Maharashtra^37^, leading to a press release on the new VoC a week later^38^. This case study suggests that the use of statistical control methods would have enabled an earlier detection of the variant.

### Emergence of the Omicron variant in South Africa

From 7 November to 4 December 2021, the Omicron variant colonized South Africa, starting with the province of Gauteng. Panels f-i of Fig. 3 show four funnel plots, where colour-coded circles represent the ${R}_{t}$’s of the South African provinces. Until the very beginning of November 2021, the Delta variant was prevalent and the differences in ${R}_{t}$ across provinces merely resulted from natural fluctuations (see panel f). By mid-November the Gauteng province crossed the upper alert threshold (see panel g) and then further diverged (see panel h). This is precisely the timing when the Omicron variant was first identified, as declared by the WHO^39^, and became a threat^40^. By 3 December 2021, Gauteng was reabsorbed within the funnel, now with a much higher mean, following the spread of Omicron in the other provinces and the consequent rise of their ${R}_{t}$’s (see panel i). The Bonferroni control chart with ± 3.09 sigma limits (panel j) clearly shows the out-of-control trajectory of the Gauteng province (red).

### Spread of the Omicron variant in England

From 4 December 2021 to 1 January 2022, the Omicron variant massively spread in England. Panels k-n of Fig. 3 show four funnel plots, with colour-coded circles corresponding to the ${R}_{t}$’s of the English regions . On December 4, all the regions were within the alarm limits (panel k). By 10 December 2021, the London region had crossed the funnel limits (panel l), further diverging from the upper limit on 15 December (panel m). This suggests that Omicron was more prevalent in London than in the rest of England and indeed, on 13 December 2021, 20% of the cases in England and over 44% of the cases in London were attributed to Omicron^28^. As the other regions were colonized, the distribution of their ${R}_{t}$’s moved upward and, on 23 December 2021, the London region was again inside the funnel (panel n). An earlier detection would have been allowed by the Bonferroni control chart, where London first crossed the alarm limit in early December (panel o).

### Immensa scandal in England

Our last case study concerns England in the period from 27 August to 25 September 2021. Panels a-d of Fig. 4 display four funnel plots in selected dates, with colour-coded circles corresponding to the ${R}_{t}$’s of the English regions. On 5 September 2021, all English regions were within the funnel (panel a). By 9 September 2021, the South West (red) had crossed the lower alarm limit (panel b) and remained below the lower limit for about two weeks (panel e). The timing of this swing coincides with the period during which the Immensa lab in Wolverhampton gave some 43,000 incorrect negative tests relative to South West and West Midlands territories^30,31^. While the suspension of lab operations came in mid-October, the Bonferroni control chart indicated an out-of-control condition already in early September and would have allowed a much earlier detection of the anomaly.

### Trajectory plots and funnel movies

**Figure 5:**
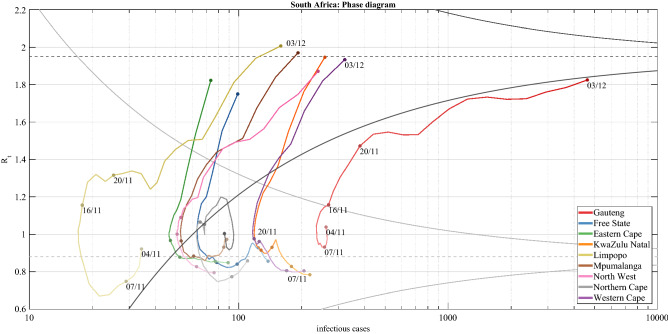
Moving funnels: the effect of the Omicron variant on the R_t_ distribution in South Africa. The figure displays the joint trajectories of infectious cases and R_t_’s of the South African provinces from 4 November 2021 to 3 December 2021, with colours getting darker over time. The x-axis scale is logarithmic to improve readability. The funnel plots of 4 November 2021 (grey) and 3 December 2021 (black) are plotted with their mean (dashed) and alarm limits (continuous). In the first date, before the spread of Omicron, the average R_t_ was below 1 and all points were inside the grey funnel plot. Then, Gauteng’s R_t_ (red) moved upwards, followed by the other provinces. Overall, Omicron caused an upward escape of the province where it first became dominant (Gauteng, red line), followed by a collective drift of the R_t_’s of other provinces, until a new funnel, i.e., the black one, was established at a higher level. The trajectories go leftwards when R_t_ is less than one, because the infectious cases tend to decrease, while the trajectories go rightwards when R_t_ is greater than one. Therefore, the trajectories exhibit a characteristic clock-wise trend^41^. Supplementary {2} describes in detail how to obtain an animated representation of this phenomenon by iteratively updating the R_t_’s and the funnels shape, leading to the “funnel movies” available in Supplementary {3}.

A better understanding of the time evolution of regional ${R}_{t}$’s is achieved when complementing funnel plots with the time dimension. This can be done in two ways. The first approach is to display the trajectories of the regional ${R}_{t}$’s: an example relative to South Africa is provided in Fig. 5. Alternatively, the funnel plot can be animated, thus yielding a “funnel movie”, where both the trajectories and the shapes of the funnels are iteratively updated (see Supplementary {2} for the description and {3} for the movies). As observed in^41^, when ${R}_{t}$ is plotted against the number of infective subjects, the trajectories exhibit a peculiar clock-wise spiral-shaped pattern. An analogous behavior was observed also in [^42^, Fig. 2], where trajectories were plotted in the plane of infected cases against “cooperators”, a variable connected with ${R}_{t}$.

### Monitoring regional ${{\varvec{R}}}_{{\varvec{t}}}$’s

In addition to the Bonferroni control charts, a further monitoring and visualization tool is obtained by plotting the regional ${R}_{t}$’s along with the national ${R}_{t}$ and with control limits defining the in-control band for the specific region. A distinct plot is needed for each region, because the width of the in-control band depends on the number of the infectious subjects in that region. Hence, differently from the Bonferroni control chart previously introduced, as many plots are needed as the number of regions. As an advantage, this visualization provides a direct display of the regional ${R}_{t}$ and may therefore be easier to understand. Examples of these plots for Italy, England, India and South Africa are given in the Supplementary {1}.

## Discussion

We proposed funnel plots and the associated Bonferroni control chart as a valuable framework for the early detection of a new emerging or imported VoC and showed their effectiveness in six real-life scenarios based on epidemic data from Italy, India, South Africa and England. These case studies demonstrate that the proposed methodology, besides being direct and inexpensive, allows the early detection of anomalies due to different root causes, ranging from the emergence of a new VoC, and its colonization of a country, to flaws in the diagnostic system, such as the Immensa COVID-19 testing scandal in England. Once the method identifies anomalous patterns, further inquiries are needed to assess their causes.

Funnel plots provide an innovative and statistically rigorous tool for monitoring the statistical homogeneity of the distribution of regional ${R}_{t}$’s. Our method can be seen as an extension to epidemiology of the funnel charts advocated by Spiegelhalter in the assessment and comparison of institutional performances in the healthcare sector^17^. Before then, funnel plots were mainly known as a standard tool for investigating biases in meta-analysis studies. As such, they have also been employed in the context of COVID-19 meta-analyses, see e.g.,^43^.

Prompt identification of a VoC before its large-scale spread, leading to impactful public health implications, is a key goal in the control of the SARS-CoV-2 pandemic and in preventing and controlling future pandemics. However, as the relentless and flashy worldwide dissemination of the Omicron variant has largely proven, some doubts remain about the most effective way to achieve this goal. Although some rRT-PCR–based algorithms and/or NAAT-based screening assays have been proposed for the early identification of VoCs^44,45^ and might be implemented in routine laboratories^46^, Whole Genome Sequencing, or at least the complete or partial sequencing of the spike (S) protein-gene, remains the only tool to both effectively identify the different variants and follow the evolution of SARS-CoV-2^47,48^. However, WGS is time consuming, expensive, and needs dedicated structures and personnel with technical expertise to be timely implemented. Furthermore, it is challenging to be applied on low viral loads samples^49^.

Exactly in this breach, the potential support of surveillance based on funnel plots and Bonferroni charts might accelerate the detection of a new VoC, without requiring, at least initially, the backup of a specialized microbiology laboratory. The value of WGS is undisputed, but, in a setting with limited resources, easy and inexpensive data-driven statistical methodologies for surveillance may support more targeted and focused genomic sequencing. Therefore, besides being extremely useful where sequencing is lacking due to scarce resources, the funnel plot framework is also precious to inform and suggest where sequencing efforts should be concentrated. It also allows the detection of anomalies that cannot be revealed by sequencing, such as failures of the testing infrastructure, as shown by the Immensa case study.

The statistical underpinning of the methodology takes into account the natural variability of the phenomenon, thus preventing false alarms even in the presence of noisy data, e.g., due to late registration of new cases. While polished data may be available with weeks of delay, funnel plots can work in real-time using the latest data, a crucial feature to allow an early detection of anomalies and hence prompt interventions. For instance, the Italian funnel plots of the first case study were fed by daily published unprocessed data.

Other authors have proposed the application of statistical process control methods for monitoring the evolution of the COVID-19 pandemic. For instance^50^, proposed hybrid control charts to detect the start and end of exponential growth in reported deaths within a geographic area. An interesting use of hybrid control charts was investigated in^51^, keeping under control exponential and non-exponential growth and decline of cases, disaggregated at the regional and subregional level, to inform local mitigation and containment strategies. Conversely, our approach leverages the characterization of the collective distribution of regional ${R}_{t}$’s: we do not monitor each region individually, but rather surveil the homogeneity of the distribution.

In view of its nature, the proposed method reveals the loss of statistical stability, but cannot of course unravel its cause. Consistently with established quality control practices, it should be used to trigger an inspection. Therefore, the funnel plot is not a VoC-detector, but an anomaly detector: early detection enables focused inquiries aimed at discovering the cause of the anomaly. In funnel plots, a point lying outside the funnel limits is associated with high confidence to some anomaly of the effective reproduction number. This may be due to several special causes of variations, such as VoCs, outbreaks due to violations of containment measures, failures of the diagnostic infrastructure (such as the Immensa scandal). In the absence of special causes, all funnel plots in the paper are designed so that all points are inside the alarm limits in 99.8% of the cases (or, equivalently, so that the false alarm probability is 0.2%).

## Methods

### Data

Data regarding new positive cases were obtained from publicly available sources: https://github.com/pcm-dpc/COVID-19/tree/master/dati-regioni for Italian data, https://data.covid19bharat.org/ for Indian data, https://mediahack.co.za/datastories/coronavirus/data/# for South African data, https://coronavirus.data.gov.uk/details/download for English data.

Following^52^, we assumed a discretized lognormal distribution for the serial interval, with parameters chosen in accordance with^53^. To correct systematic errors in the data, partly due to the weekly periodicity, partly due to delays and other reporting errors, all data were filtered using a double seven-day moving average.

### Funnel plots and Bonferroni control charts

In a funnel plot, a measured or estimated quantity is plotted against an interpretable measure of its precision. A funnel plot is composed of four elements^17^: (i) an indicator $Y$ that represents the quantity to be monitored, (ii) a reference value $\theta$ that specifies the expectation of the indicator, (iii) a precision parameter $\rho$ that determines the accuracy with which the indicator is measured, (iv) the control limits ${y}_{lower}$, ${y}_{upper}$ that specify the boundaries of the out-of-control region. An example of funnel plot can be seen in Fig. 1. The dot ${(\rho }_{i},{y}_{i})$ is associated with the $i$-th region, where ${\rho }_{i}$ is the number of infectious cases in the region and ${y}_{i}$ is the region’s reproduction number ${R}_{t}$ at a given time $t$. The horizontal line $Y=\theta$ shows the national average ${R}_{t}$ and the funnel-shaped pair of control limits ${y}_{lower}$ and ${y}_{upper}$ shows where we would expect the regions to lie if their ${R}_{t}$’s were statistically indistinguishable from one another, see Panel d in Fig. 1.

In several circumstances, an exact or approximate normal distribution of the indicator $Y$ can be assumed

1 $$Y|\theta ,\rho \sim N\left[ {\theta ,g\left( \theta \right)/\rho } \right]$$

where $g$ is a suitable function of $\theta$^17^ such that $Var[Y]=g(\theta )/\rho$. Under this null hypothesis, with probability $1-\alpha$,

$$\theta - z_{{\frac{\alpha }{2}}} \sqrt {\frac{g\left( \theta \right)}{\rho }} \le Y \le \theta + z_{{\frac{\alpha }{2}}} \sqrt {\frac{g\left( \theta \right)}{\rho }}$$

where ${z}_{\mathrm{\alpha }/2}$ is such that $P\left(Z\le {z}_{\mathrm{\alpha }/2} \right)=1-\frac{\alpha }{2}$ for a standard normal variable $Z$. For instance, ${z}_{\mathrm{\alpha }/2} =1.96$, when $\alpha =5\%$, and ${z}_{\mathrm{\alpha }/2} =3.09$, when $\alpha =0.2\%$. This means that, in $100\left(1-\alpha \right)\%$ of the cases, $Y$ is expected to lie within the lower and upper control limits defined as

$$y_{lower} = \theta - z_{\alpha /2} \sqrt {g\left( \theta \right)/\rho }$$

$$y_{upper} = \theta + z_{\alpha /2} \sqrt {g\left( \theta \right)/\rho }$$

By introducing the Z-score

$$z_{i} = \frac{{y_{i} - \theta }}{{\sqrt {g\left( \theta \right)/\rho } }}$$

we have that $P({|z}_{i}|\le {z}_{\alpha /2})=1-\alpha$. In Statistical Process Control, the common practice is to select a false alarm probability as small as $\alpha =0.2\%$, corresponding to ${z}_{\alpha /2}\ge 3.09.$ A Z-score whose absolute value is greater than ${z}_{\alpha /2}$ is said to be *out of (statistical) control* and deemed worthy of study to identify a special cause of variation that explains its departure from the mean. Note that there is a $0.2\%$ probability of reporting an out-of-control point when no special cause of variation is actually perturbing the process and the outlier arises by pure chance under common causes of variation.

When monitoring $n$ units of analysis, e.g., the ${R}_{t}$ of $n$ regions within a country, due to the multiple comparison problem, the false positive rate could become unacceptably large. A simple way to address this problem is the Bonferroni correction that replaces $\alpha$ with $\alpha /n$^54^.

When the indicators ${y}_{i}$ measure a frequency of occurrence, e.g., the mortality rates in heart surgery units, it is reasonable to assume a binomial model, with $\theta$ representing the probability of the event and ${\rho }_{i}$ the number of surgeries in the $i$-th unit. For the binomial model, the variance of ${y}_{i}$ is $\theta (1-\theta )/{\rho }_{i}$ so that, given $\theta$, the variance of ${y}_{i}$ is completely specified. For a large enough $\rho$, the binomial converges to a normal random variable that follows distribution (1) with $g(\theta )=\theta (1-\theta )$. An analogous case is when the products ${\rho }_{i}{y}_{i}$ are Poisson distributed with expectation ${\rho }_{i}\theta$. If ${\rho }_{i}\theta$ is greater than 30, the indicators ${y}_{i}$ are then normally distributed as (1) with $g(\theta )=\theta .$ Therefore, for both the ideal binomial and Poisson model, estimating the mean of ${y}_{i}$ suffices to specify both the centerline and the alarm limits of the funnel plot.

However, as discussed in^18^, if one lets the variance be specified by the mean, it very often happens that the fraction of units of analysis that lie outside the ideal alarm limits greatly exceeds the theoretical false positive rate. This phenomenon, well known in the statistical literature, goes under the name of *overdispersion*^18^. This can be dealt with by modifying (1) with the introduction of an overdispersion parameter $\phi$ to be estimated from data:

2 $$Y|\theta ,\rho \sim N\left[ {\theta ,\phi g\left( \theta \right)/\rho } \right]$$

The control limits and the Z-scores are redefined accordingly as

$$y_{lower} = \theta - z_{\alpha /2} \sqrt {\phi g\left( \theta \right)/\rho }$$

$$y_{upper} = \theta + z_{\alpha /2} \sqrt {\phi g\left( \theta \right)/\rho }$$

$$z_{i} = \frac{{y_{i} - \theta }}{{\sqrt {\phi g\left( \theta \right)/\rho_{i} } }}$$

When the indicators to be monitored are time series depending on a time index $t$, i.e., ${{y}_{i}=y}_{i}(t)$, a distinct funnel plot can be drawn for each time instant. For the purpose of statistical monitoring, the relevant information can be summarized in a Bonferroni control chart where the trends of the $Z$-scores are plotted in time against Bonferroni limits, see for instance panel e in Fig. 1. Under (2), we have that ${z}_{i} \sim N[\mathrm{0,1}]$, so that, when the $Z$-scores are plotted on a control chart with zero centerline and Bonferroni limits equal to ${\pm z}_{\alpha /(2n)}$, the probability of one or more dots lying outside the limits is equal to $\alpha$.

### Distribution of regional R_t_’s

The reproduction number at time $t$, named ${R}_{t}$, captures the number of secondary infections from a population including both susceptible and immune individuals. For its estimation, a range of model frameworks and estimation procedures have been proposed^55^. Herein we adopt the approach of Cori et al.^56^ that makes minimal assumptions about the mathematical model of the epidemic process. Cori’s formula uses the time series of the new cases and estimates of the distribution of the generation time, i.e., the time between infections.

According to^56^ the estimate ${\widehat{R}}_{t}$ of the instantaneous reproduction number ${R}_{t}$ is obtained as

3 $$\hat{R}_{t} = \frac{{I_{t} }}{{\mathop \sum \nolimits_{s = 1}^{t} {\textit{w}}_{{{\textit{s}}}} {\textit{I}}_{{{\textit{t}} - {\textit{s}}}} }}$$

where $I_{t}$ denotes the daily number of new infected cases and $w_{s}$ are the coefficients, adding up to one, of the infectivity profile, often approximated by the distribution of the serial interval. The denominator

$$\Lambda_{t} = \mathop \sum \limits_{s = 1}^{t} {\textit{w}}_{{\textit{s}}} {\textit{I}}_{{{\textit{t}} - {\textit{s}}}}$$

can be interpreted as the total infectiousness of individuals that are currently infected at time $t$. In view of the typical models of the infectivity profile, e.g., lognormal or gamma density functions, ${\Lambda }_{t}$ is a smoothed version of the time series ${I}_{t}$ of daily new cases. If seven-day moving averages are used to filter out weekly oscillations, ${I}_{t}$ is already smooth, and the resulting ${\Lambda }_{t}$ is insensitive to the precise shape of the infectivity profile. This feature may prove helpful when a new VoC arises whose infectivity profile is unknown, or only approximately known.

To derive the distribution of ${\widehat{R}}_{t},$ we only assume that disease transmission follows a Poisson distribution with mean ${{R}_{t}\Lambda }_{t}$:

$$I_{t} |R_{t} ,\Lambda_{t} \sim Pois\left[ {R_{t} \Lambda_{t} } \right]$$

Typically, $R_{t} \Lambda > 30$, so that a normal approximation can be used:

$$I_{t} |R_{t} ,\Lambda_{t} \sim N\left[ {R_{t} \Lambda_{t} ,R_{t} \Lambda_{t} } \right]$$

In view of (3), it follows that ${\widehat{R}}_{t}|{{R}_{t},\Lambda }_{t}\sim N[{R}_{t},{R}_{t}/{\Lambda }_{t}]$. For the sake of interpretability, rather than using the notion of total infectiousness ${\Lambda }_{t}$, it is more intuitive to refer to the total number of infectious individuals. To this aim, we introduce the parameter

$$\gamma = \frac{1}{{\mathop \sum \nolimits_{s = 1}^{\infty } sw_{s} }}$$

i.e., the inverse of the mean serial interval, which, for the well-known SIR model, corresponds to the removal rate^57^. Then, ${\rho }_{t}={\Lambda }_{t}/\gamma$ represents the number of individuals that are infectious at time $t$. Letting $\theta ={R}_{t}, g(\theta )={R}_{t}/\gamma$, it follows that

$$\hat{R}_{t} |\theta ,\rho_{t} \sim N\left[ {\theta ,g\left( \theta \right)/\rho_{t} } \right]$$

Comparing the above distribution with (1), it follows that, for any given $t$, the scatter plot of ${\widehat{R}}_{t}$ against ${\rho }_{t}$ is indeed a funnel plot. Also in this case, it is convenient to introduce an overdispersion parameter $\phi$, so that, in accordance with (2), the final model becomes

4 $$\hat{R}_{t} |\theta ,\rho_{t} \sim N\left[ {\theta ,\phi g\left( \theta \right)/\rho_{t} } \right]$$

A useful byproduct of introducing overdispersion is that $\phi$ takes into account the effect that possible errors or uncertainties in the estimated mean serial interval has on the variance of ${\widehat{R}}_{t}$. Indeed, the variance of ${\widehat{R}}_{t}$ is inversely proportional to $\gamma$, but the effect of a wrong $\gamma$ is automatically compensated when estimating $\phi$ from the data.

### Parameter estimation

Under (2), the estimated reproduction number ${\widehat{R}}_{t}^{i}$ of the $i$-th region can be written as

5 $$Y_{i} = \theta + \frac{{\varepsilon_{i} }}{{\sqrt {x_{i} } }}$$

where ${Y}_{i}={\widehat{R}}_{t}^{i}$, ${x}_{i}={\rho }_{t}^{i}$ is the number of infectious individuals, and ${\varepsilon }_{i} \sim N\left[0,{\sigma }^{2}\right]$, $i=1,\dots ,n$, are mutually independent with ${\sigma }^{2}=\phi g(\theta )$. Letting ${v}_{i}={\varepsilon }_{i}/\sqrt{{x}_{i}}$, the model in matrix form becomes

$$Y = \Phi \theta + v$$

where $$Y = \left[ { \ldots Y_{i} \ldots } \right]^{^{\prime}}$$, $$\Phi = \left[ { \ldots 1 \ldots } \right]^{^{\prime}}$$, $$v\sim N\left[ {0,\sigma^{2} \Sigma } \right]$$$$,$$ and

$$\Sigma = \left( {\begin{array}{*{20}c} {\frac{1}{{x_{1} }} \cdots 0 } \\ { } \\ { \vdots \ddots \vdots } \\ \\ { 0 \cdots \frac{1}{{x_{n} }} } \\ \end{array} } \right)$$

Then, the generalized least squares technique^58^ provides the minimum variance unbiased estimate and the estimated parameters are

$$\hat{\theta } = \left( {\Phi^{\prime}\Sigma^{ - 1} \Phi } \right)^{ - 1} \Phi ^{\prime}\Sigma^{ - 1} Y$$

6 $$\widehat{{\sigma^{2} }} = \frac{1}{n} e^{\prime}\Sigma^{ - 1} e$$

where $e=Y-\Phi \widehat{\theta }$ is the vector of the residuals. Recalling that $g(\theta )=\theta /\gamma$, the overdispersion parameter $\phi$ is estimated as $\widehat{\phi }= \gamma \widehat{{\sigma }^{2}}/\widehat{\theta }$. Data winsorization can be performed, as detailed in^17^, to reduce the effect of possibly spurious outliers.

Estimates $\widehat{\theta }$ and $\widehat{\phi }$ are needed to compute the funnel at time $t$, as well as the standardized residuals ${z}_{i}$ to be plotted in the Bonferroni control chart. The estimate $\widehat{\phi }$ is computed from (6) using the set of ${\widehat{R}}_{t}^{i}$ that were in control at time $t-1$. The centerline $\widehat{\theta }$ is obtained by projecting at time $t$ a weighted linear regression estimated from the set of ${\widehat{R}}_{t}^{i}$ that were in control at times $t-1, t-2,$ and $t-3$. The weights are given by the numbers ${x}_{i}$ of infectious individuals at the same times. This procedure yields an estimate of the current $\theta$ that tracks the trends of the national ${R}_{t}$, but is still fairly robust thanks to the use of the last three data points. At the beginning (and in the rare cases when all units are out of control), the whole sets $\left\{{\widehat{R}}_{t}^{i}\right\}, \left\{{\rho }_{t}^{i}\right\}$ are fed to the estimator.

### Ethics declarations

No ethics review and informed consent/animal welfare protocols were needed, because the research entirely relied on epidemiological time series that are publicly accessible.

## Supplementary Information

Supplementary Information 1.

Supplementary Information 2.

Supplementary Information 3.

## Data Availability

Data regarding new positive cases can be obtained from publicly available sources: https://github.com/pcm-dpc/COVID-19/tree/master/dati-regioni for Italian data; https://data.covid19bharat.org/ for Indian data; https://mediahack.co.za/datastories/coronavirus/data/# for South African data; https://coronavirus.data.gov.uk/details/download for English data.

## Code availability

Code will be made available online.
