# Supplementary material for: Early detection of variants of concern via funnel plots of regional reproduction numbers
Source: Sci Rep. 2023 Jan 19;13:1052. doi: 10.1038/s41598-022-27116-8 (PMC9852294; doi:10.1038/s41598-022-27116-8)
Supplement: Supplementary file 1 — Supplementary Information 1. [file 41598_2022_27116_MOESM1_ESM.docx]

**Supplementary information {1}**

**Early detection of Variant of Concern via funnel plots of regional reproduction numbers**

S. Milanesi, F. Rosset, M. Colaneri, G. Giordano, K. Pesenti, F. Blanchini,
P. Bolzern, P. Colaneri, P. Sacchi, G. De Nicolao, R. Bruno

*Individual* $R_{t}$ *charts*

As an alternative to the Bonferroni control chart, one can resort to a set of individual charts for the regional $R_{t}$'s, equipped with control limits around the national $R_{t}$, delimiting the in-control band for each specific region. A distinct plot is needed for each region because the width of the in-control band depends on the number of the infectious subjects in that region. On the one hand, this means that, unlike the unique Bonferroni control chart, as many charts are needed as the number of regions. On the other hand, this visualization provides a direct display of the regional $R_{t}$ and may therefore be more easily understandable. In the following, South African (Figure 1), English (Figures 2, 4) and Italian (Figure 3) individual charts are displayed. The Indian charts are not shown because of the excessive number of regions. To facilitate comparison, the Bonferroni chart is also displayed in the first panel.

*Figure 1: South Africa states from November 2021 to December 2021*

Consistently with the panels f-j of Figure 3 of the paper, the $R_{t}$ of the Gauteng state exceeds the control limits in correspondence of the emergence of the Omicron variant [29]. After its exit, despite the rise of the other $R_{t}$’s, the associated states remain in-control: a new homogeneity imposed by the new variant spreading is established.

*Figure 2: English regions from December 2021 to January 2022*

Consistently with the panels k-o of Figure 3 of the paper, the $R_{t}$ of the London region exceeds the control limits in correspondence of the spreading of the Omicron variant [31, 45]. As for the South African case, despite the rise of the other $R_{t}$’s, the associated regions remain in-control: a new homogeneity imposed by the new variant spreading across the country is established.

 *Figure 3: Italian regions from December 2021 to January 2022*

Similarly to the English case (Figure 2 of this supplementary file), the $R_{t}$ of Lombardia exceeds the control limits in correspondence of the spreading of the Omicron variant [26]. This is consistent with Figure 1 of the paper.

**

*Figure 4: English regions from September 2021 to October 2021*

As depicted in the Figure 4 of the paper, the $R_{t}$ of the South West region crosses the lower limit in correspondence of the malfunctioning of Immensa laboratories [14,15].
