# Supplementary material for: Early detection of variants of concern via funnel plots of regional reproduction numbers
Source: Sci Rep. 2023 Jan 19;13:1052. doi: 10.1038/s41598-022-27116-8 (PMC9852294; doi:10.1038/s41598-022-27116-8)
Supplement: Supplementary file 2 — Supplementary Information 2. [file 41598_2022_27116_MOESM2_ESM.docx]

**Supplementary information {2}**

**Early detection of Variant of Concern via funnel plots of regional reproduction numbers**

S. Milanesi, F. Rosset, M. Colaneri, G. Giordano, K. Pesenti, F. Blanchini,
P. Bolzern, P. Colaneri, P. Sacchi, G. De Nicolao, R. Bruno

*Funnel movies*

The main limit of funnel plots is that they cannot capture the time evolution of the regional $R_{t}$’s: the funnel plot is a static picture of a fixed day. If we want to gain a better understanding of the evolution of the $R_{t}$'s monitoring, there are two possible ways to proceed. The first approach displays the trajectories of the regional $R_{t}$'s and an example relative to South Africa is provided in Figure 5 of the paper. Alternatively, the funnel plot can be animated yielding a “funnel movie”, where both the trajectories and the shapes of the funnels are iteratively updated (see Supplementary {3} for the videos associated to Italian, South African, Indian and English case studies). As one can verify in the movies, a typical behavior occurs: the trajectories go leftwards when R_t_ is less than one, because the infectious cases tend to decrease, while the trajectories go rightwards when R_t_ is greater than one. Therefore, the trajectories exhibit a characteristic clockwise pattern. This feature has a prognostic value: to have a slowdown, you have to wait for the trajectory to become horizontal and then fall.
